# Typhoid fever in Fiji: a reversible plague?

**DOI:** 10.1111/tmi.12367

**Published:** 2014-07-25

**Authors:** Corinne N Thompson, Mike Kama, Shrish Acharya, Una Bera, John Clemens, John A Crump, Aggie Dawainavesi, Gordon Dougan, W John Edmunds, Kimberley Fox, Kylie Jenkins, M Imran Khan, Josefa Koroivueta, Myron M Levine, Laura B Martin, Eric Nilles, Virginia E Pitzer, Shalini Singh, Ratu Vereniki Raiwalu, Stephen Baker, Kim Mulholland

**Affiliations:** 1 Hospital for Tropical Diseases, Wellcome Trust Major Overseas Programme, Oxford University Clinical Research UnitHo Chi Minh City, Vietnam; 2 Centre for Tropical Medicine, Nuffield Department of Clinical Medicine, Oxford UniversityOxford, UK; 3 Fijian Ministry of HealthSuva, Fiji; 4 Colonial War Memorial HospitalSuva, Fiji; 5 UCLA School of Public HealthLos Angeles, CA, USA; 6 Centre for International Health, University of OtagoDunedin, New Zealand; 7 World Health Organization Division of Pacific Technical SupportSuva, Fiji; 8 Wellcome Trust Sanger InstituteHinxton, Cambridgeshire, UK; 9 London School of Hygiene and Tropical MedicineLondon, UK; 10 World Health Organization Regional Office for the Western PacificManila, Philippines; 11 Fiji Health Sector Support ProgramSuva, Fiji; 12 International Vaccines InstituteSeoul, Korea; 13 University of Maryland School of MedicineBaltimore, MD, USA; 14 Novartis Vaccines Institute for Global HealthSiena, Italy; 15 Yale School of Public HealthNew Haven, CT, USA; 16 Menzies School of Health Research, Royal Darwin Hospital CampusDarwin, NT, Australia

**Keywords:** typhoid, Fiji, enteric fever, transmission, epidemiology

## Abstract

The country of Fiji, with a population of approximately 870 000 people, faces a growing burden of several communicable diseases including the bacterial infection typhoid fever. Surveillance data suggest that typhoid has become increasingly common in rural areas of Fiji and is more frequent amongst young adults. Transmission of the organisms that cause typhoid is facilitated by faecal contamination of food or water and may be influenced by local behavioural practices in Fiji. The Fijian Ministry of Health, with support from Australian Aid, hosted a meeting in August 2012 to develop comprehensive control and prevention strategies for typhoid fever in Fiji. International and local specialists were invited to share relevant data and discuss typhoid control options. The resultant recommendations focused on generating a clearer sense of the epidemiology of typhoid in Fiji and exploring the contribution of potential transmission pathways. Additionally, the panel suggested steps such as ensuring that recommended ciprofloxacin doses are appropriate to reduce the potential for relapse and reinfection in clinical cases, encouraging proper hand hygiene of food and drink handlers, working with water and sanitation agencies to review current sanitation practices and considering a vaccination policy targeting epidemiologically relevant populations.

## Introduction

Enteric fever is a systemic, human-restricted infection caused predominantly by *Salmonella enterica* serovars typhi (*S. typhi*) and Paratyphi A (*S. paratyphi* A), resulting in typhoid and paratyphoid fevers, respectively (Parry *et al*. 2002; Basnyat 2005). Transmitted faecal orally through contaminated food or water, typhoid is generally associated with poor sanitation and hygiene (Swaddiwudhipong & Kanlayanaphotporn 2001; Kothari *et al*. 2008). Bacteraemia presents after 1–2 weeks, causing a persistent fever with malaise and can result in protracted illness lasting several weeks. Although rarely fatal with appropriate antimicrobial treatment, untreated typhoid can lead to life-threatening complications including hypotensive shock and intestinal perforation (Parry *et al*. 2002; Chanh *et al*. 2004).

The Republic of Fiji is a country in the South Pacific with a population of approximately 870 000 people (UN 2011). The country comprises >300 islands with a total land area of 18 000 km^2^ (FMOH 2011). In addition to the challenge of providing adequate health services to a dispersed population, Fiji is faced with a substantial burden of communicable and non-communicable diseases as it undergoes an economic transition to an upper-middle income country (Australian Aid 2010; FMOH 2011). The Fijian Ministry of Health (MOH) has expressed particular concern over the rapid rise in reported cases of typhoid and leptospirosis, which along with dengue fever, are currently referred to locally as the ‘three plagues’ (FMOH 2010a, 2011a; WHO/FMOH/UNDP 2011). Improved surveillance and a series of recent typhoid outbreaks have increased typhoid awareness amongst both medical professionals and the general public (Tuiketei *et al*. 2005). Reducing the incidence of typhoid and limiting outbreaks is now seen as a priority for the MOH (FMOH 2011).

## Setting and methods

In August 2012, the MOH, with support from Australian Aid, hosted a meeting of national and international typhoid fever specialists in an effort to develop, prioritise and implement a comprehensive control and prevention strategy for typhoid in Fiji. This article summarises the known epidemiology, clinical characteristics and microbiological trends of typhoid and highlights corresponding gaps in practice and policies relevant to the disease in Fiji. This epidemiological evaluation was conducted using available published data, MOH documents and information from interviews with members of the local public health, medical, and water and sanitation communities. Data regarding all laboratory-confirmed *S. typhi* cases spanning January 2008–August 2012 were provided by the Fiji Centre for Communicable Disease Control**,** Mataika House, Fiji MOH. Data were cleaned and imported into STATA v11 (StataCorp, TX, USA), and descriptive analyses on age, sex, ethnicity and geographic location were performed. A summary and brief description of the recommendations developed during the meeting are also presented and discussed in the context of the realities of disease control in Fiji.

## The epidemiology of typhoid in Fiji

Some South Pacific islands have been known to experience high typhoid fever incidence rates and outbreaks. For example, a prospective, population-based study conducted in rural Papua New Guinea in the mid-1990's found an incidence of culture-positive typhoid fever of 817/100 000 population (Talme *et al*. 1994; Passey 1995). Additional available reports from the region include outbreaks in Nauru in the Central Pacific and in Western Samoa after cyclones Ofa and Val in 1990–93 (SDOH 1993; Olsen *et al*. 2001). Recent data from other low- and middle-income countries in the region are limited (Olsen *et al*. 2001; Dunn *et al*. 2005).

### Incidence and recent trends

Typhoid fever is endemic in Fiji, and there has been a steep increase in reported cases over the last decade (FMOH 2010a). Prior to 2005, the incidence of reported culture-confirmed cases of typhoid was generally <5/100 000 population (Dunn *et al*. 2005; Tuiketei *et al*. 2005). However, this figure increased to 33/100 000 in 2005 (Dunn *et al*. 2005) and climbed to 44, 40, 52 and 42/100 000 in 2008, 2009, 2010 and 2011, respectively (Scobie *et al*. 2014*)*. Explanations for this sudden increase from 2005 may include better surveillance and diagnostics, improved clinician awareness and/or an actual increase in caseload (Dunn *et al*. 2005; Tuiketei *et al*. 2005; FMOH 2010a; Jenkins 2010).

**Figure 1:**
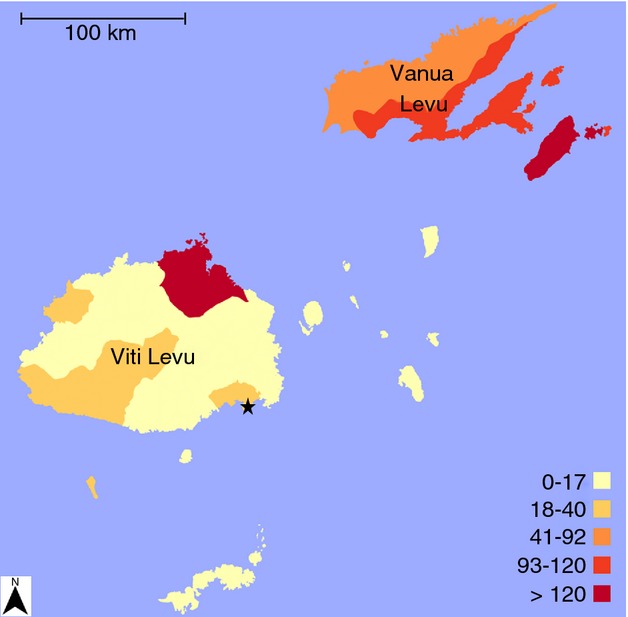
Average annual laboratory-confirmed incidence of *S. Typhi* cases per 100 000 population in Fiji by subdivision, January 2008–August 2012. Incidence per 100 000 population is indicated by colour, with darker colours reflecting higher incidence. The capital of Fiji, Suva, is shown by the black star.

Available clinical and laboratory-confirmed surveillance data suggest that Fiji experiences a peak of typhoid cases between January and June annually, corresponding approximately with the peak of the rainy season that extends from November to April (WHO/FMOH/UNDP 2011; Scobie *et al*. 2014). The distribution of reported cases of typhoid in Fiji varies dramatically geographically, although it is generally considered a more substantial problem in rural areas (FMOH 2010a). The main island of Viti Levu is comprised of the Central and Western Divisions and has several large coastal cities and a sparsely populated interior (Figure1). The second largest island (Vanua Levu) comprises the majority of the Northern Division and has historically reported both a higher incidence and a higher absolute number of typhoid cases than the other divisions (Dunn *et al*. 2005). In 2008, the estimated incidence of reported culture-confirmed cases per 100 000 population was 176, 22 and 19 in the Northern, Western and Central divisions, respectively (Scobie *et al*. 2014). It has been suggested anecdotally that typhoid has been endemic for a considerable time on Vanua Levu, yet is relatively new on Viti Levu (Jenkins 2010; Scobie *et al*. 2014) (Figure1). However, disease patterns are difficult to characterise accurately as major gaps remain in typhoid surveillance.

### Case description

**Figure 2:**
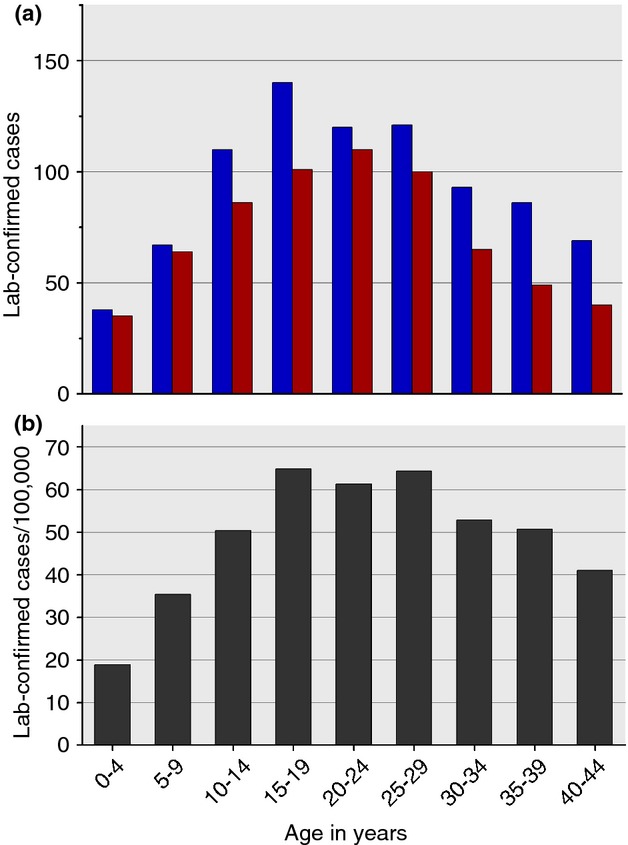
Age distribution of laboratory-confirmed typhoid fever cases in Fiji, 2008–2011. (a) Total number of reported cases by age and sex, blue: male cases; red: female cases. (b) The age-specific incidence per 100 000 population.

Of the 1847 culture-confirmed typhoid cases reported between January 2008 and July 2012, the median (range) age was 25 (0–95) years (Figure2a) and 1043 (57%) were male, which is consistent with previous reports from Fiji (Dunn *et al*. 2005; Scobie *et al*. 2014). The highest laboratory-confirmed incidence (average: 64/100 000 population) was among 15- to 29-year-olds (Figure2b). High incidence among young adults is similar to previous reports from Fiji (Dunn *et al*. 2005; Scobie *et al*. 2014), although contrasts with other regions endemic for typhoid where incidence generally peaks among young and school-aged children (Ochiai *et al*. 2008). However, rigorous population-based studies of the age distribution of typhoid fever suggest that the average age of typhoid cases appears to fall as incidence rates increase; a peak of cases among young adults is consistent with a medium incidence setting (Crump *et al*. 2004). However, there may also be age-related diagnosis and reporting biases present as laboratory confirmation in young patients may be hampered by a low blood culture volume (FMOH 2010a), differences in clinical presentation by age and the notoriously challenging non-specific symptoms in this age group (Bhutta 2006). This is further compounded by inconsistent case definitions for paediatric typhoid, which differ among hospitals in Fiji and make a combined analysis of typhoid in children of little value.

The population of Fiji is composed of both indigenous Fijians (57%, predominately Melanesian) and Fijians of Indian ancestry (38%) who descend largely from Indian contract labourers brought to the islands by the British in the 19th century (FBOS 2007). Typhoid appears to affect primarily indigenous Fijians, with >90% of all reported cases occurring in this group (Dunn *et al*. 2005; FMOH 2010a; Singh 2010; Scobie *et al*. 2014). The reasons behind the association with typhoid and ethnic group are unclear. It is possible that cultural and behavioural practices may influence the transmission of typhoid in this setting (Singh 2010). Alternatively, the 2007 census shows Fijians of Indian ancestry are more likely than indigenous Fijians to live in urban areas (57% *vs*. 44%, respectively) (FBOS 2007), where the prevalence of typhoid is thought to be lower (FMOH 2010a). Additionally, Fijians of Indian ancestry may have different health-seeking behaviour and consequently may present to private general practitioners before typhoid fever is suspected and/or able to be cultured (Singh 2010). Although such private practices are required to report suspected cases of typhoid to the MOH (fever ≥3 days with severe headache, abdominal pain, diarrhoea or constipation; recent travel; case contact) (PHA 2013), compliance may be limited.

### Transmission

Epidemiologic research on the patterns and mechanisms of typhoid transmission in Fiji has not been performed, and no risk factors have been investigated or substantiated. However, transmission studies have been performed in other countries in the region, with consumption of fish contaminated with sewage suggested as the source of an outbreak in Nauru, for example (Olsen *et al*. 2001). A hypothesis amongst some in the Fijian public health community is that transmission of *S*. *typhi* takes place largely through the consumption of a popular drink known as *kava* or *yaqona*. *Kava* is made by grinding the root of the plant *Piper methysticum* and mixing it with water by hand. It has mild anaesthetic and anxiolytic properties (Sarris *et al*. 2013), and is consumed socially, mostly among men who gather and share one bowl. Traditionally, indigenous Fijian men are most likely to consume *kava*, but sometimes women and Fijians of Indian ancestry are invited to share a bowl. Additional hypotheses of transmission include poor personal hygiene and sanitation, particularly in the context of large social gatherings with shared food (Kreidl 2008; FMOH 2010a). Furthermore, the Fijian population is known to have a high rate of gallbladder abnormalities (Gill 2006). As gallbladder disease is associated with becoming a chronic carrier of typhoid after infection (Levine *et al*. 1982; Parry *et al*. 2002; Gonzalez-Escobedo *et al*. 2011), there is potential for a high prevalence of carriers in the population who could act as reservoirs for infection (Parry *et al*. 2002). The true prevalence of chronic typhoid carriers in this population is unknown.

### Cyclone Tomas and the vaccination campaign of 2010

In March 2010, a category IV (winds of up to 251 km/h) tropical cyclone (cyclone Tomas) hit Fiji, resulting in substantial damage and disruption of water and sanitation in the Northern Division including Vanua Levu and Taveuni, the second and third largest islands, respectively (Jenkins 2010). In response to the cyclone, the MOH initiated a vaccination campaign using Typhim Vi (polysaccharide), targeting all residents over the age of 2 years in areas impacted by the cyclone (Scobie *et al*. 2014). Two months after the cyclone, a large typhoid fever outbreak occurred in the central highlands of Viti Levu, the largest island (which was largely unaffected by the cyclone), with 29 culture-confirmed cases and an additional 250 suspected cases. Until this outbreak, typhoid had not been previously reported in this area (Jenkins 2010). Therefore, vaccine procured for the preventive post-disaster response was used reactively in the outbreak areas as well (Scobie *et al*. 2014). Scobie and colleagues recently published an evaluation of this large-scale Vi-polysaccharide vaccine intervention, the first of its kind in a post-disaster setting in the Pacific (Scobie *et al*. 2014). Between June and December 2010, approximately 70 000 doses of vaccine were administered to all individuals ≥2 years of age in various parts of the country; coverage of the target population in regions of the vaccination campaign ranged from 84% to over 100% (due to discrepancies in population present and census data) (Scobie *et al*. 2014). Approximately 7% of the total Fijian population was vaccinated during this campaign (Scobie *et al*. 2014). Further evaluation of the campaign is ongoing.

## Clinical characteristics & microbiological trends

Physicians in Fiji reported an increase in the number of clinical typhoid fever cases among patients seen in clinics from 2005 onward, lending credibility to an actual increase in incidence of typhoid from that period (Dr Shrish Acharya, Colonial War Memorial Hospital, personal communication). However, the clinical presentation of typhoid is variable and overlaps with other febrile conditions, making an accurate diagnosis difficult. Culture-confirmed typhoid case fatality rates remain low in Fiji (Tuiketei *et al*. 2005). Yet it is estimated that 60–90% of typhoid patients do not get medical treatment or are treated as outpatients (Tuiketei *et al*. 2005; Kreidl 2008), complicating generalisability of hospital-based fatality rates.

The sale of antimicrobials is generally well controlled in both hospitals and the private sector in Fiji. Chloramphenicol was the first-line antimicrobial for the treatment of typhoid in Fiji until 2010 when the MOH recommended ciprofloxacin as the treatment of choice for all patients (except pregnant women) (FMOH 2010b). Treatment with ciprofloxacin was implemented to reduce the potential for long-term carriage and to shorten the treatment course to improve patient adherence (FMOH 2010a). Although MOH guidelines state the optimal ciprofloxacin dose is 15 mg/kg with a maximum of 30 mg/kg daily for 5 days (FMOH 2010b), complying with World Health Organization (WHO) recommendations (WHO 2003), the standard ciprofloxacin dosage for adults at the main hospital in the capital Suva is 500 mg twice daily. This dose is appropriate within the MOH guidelines for patients weighing 33–66 kg. The average weight of adult males and female Fijians, however, has been shown to be greater than this (90 and 70 kg, respectively) (Brian *et al*. 2011), suggesting that there may be a level of under-dosing in hospitalised patients, particularly in adult males. Although side effects of increased dosing of ciprofloxacin are possible, the minimum dose of 15 mg/kg for a 90-kg person is still within recommended dosing strategies for conditions such as bone and skin infections (FDA 2004).

Isolation of *Salmonella* is performed at three hospitals in Fiji, all of which have trained microbiology staff with automated microbial detection and antimicrobial susceptibility testing capacity. The microbiology laboratories aim to identify bloodstream infection in suspected typhoid patients by inoculating blood in tryptone soya broth and sodium polyethanol sulphonate, or to isolate *Salmonella* from other sites by plating stool, rectal swabs, urine or gallbladder aspirates directly onto MacConkey and/or Xylose Lysine Deoxycholate (XLD) agar plates. On observation of turbidity in blood cultures, they are subcultured onto MacConkey and other media. Suspected *Salmonella* from MacConkey and/or XLD agar plates are distinguished from other Gram-negative bacteria using the Microbact A&B system, a biochemical short-set (triple sugar iron) and lysine indole motility. Further identification is performed by serogrouping; all suspected invasive *Salmonella* are subjected to slide agglutination to identify Group A (O2 – suspected *S.* Paratyphi A), Group D (O9 – suspected *S. typhi*) and Vi positive (*S. typhi*) *Salmonella* (Singh 2010). *S.* Paratyphi A is very rarely identified in Fiji (Dunn *et al*. 2005).

Currently, resistance to antimicrobials in *S. typhi* is uncommon in Fiji. From a report examining 441 cases of *S. typhi* infection from 2000 to 2010 from the Central/Eastern Division, none of the isolates were resistant to any of the antimicrobials tested (including cephalosporins and fluoroquinolones) (Singh 2010). This low prevalence of antimicrobial resistance confirms previous findings (Dunn *et al*. 2005) and suggests that the global epidemic caused by *S. typhi* strains with reduced susceptibility to fluoroquinolones has not, as yet, reached Fiji (Chau *et al*. 2007).

## Current policy and practice gaps

### Surveillance and reporting

The rapid identification of typhoid cases is critical for the control and treatment efforts of the Fijian MOH. There are currently two typhoid surveillance systems in Fiji: one based on clinical reporting and the other based on culture-confirmed cases. The lack of any sensitive and specific clinical characteristics to distinguish among typhoid fever, leptospirosis and dengue fever limits the reliability of clinical diagnoses (WHO/FMOH/UNDP 2011). Timeliness of reporting and high levels of incomplete documentation are consistent issues affecting typhoid reporting and surveillance in Fiji, making evaluation of the dynamics of typhoid in this country difficult (Saunders 2001; Dunn *et al*. 2005). An additional limitation is the lack of reporting by private practitioners (Kreidl 2008). As outbreaks of typhoid continue to occur in Fiji, in addition to ongoing endemic disease, upgrading the surveillance system will be important to identify sources of infection, prevent additional outbreaks and monitor the epidemiology of typhoid to better inform policy development (Koroivueta *et al*. 2010).

### Water & sanitation infrastructure

Fiji and other Pacific island countries face many challenges in providing access to safe drinking water. These challenges include the constant threat of natural disasters, poor water supply infrastructure and highly variable abundance of water resources (Dupont 1986; Sem & Underhill 1992; Duncan 2011). According to the United Nations Children's Fund (UNICEF) and WHO, 97% of urban Fijians have access to improved piped water compared with 66% in rural areas (WHO/UNICEF 2012). However, despite the existence of water quality standards (FMOH 2006), urban water supplies are not generally considered to be microbiologically safe due to lack of enforcement (David Duncan, Applied Geoscience and Technology Division of the Secretariat of the Pacific Community [SOPAC], personal communication). A secondary and important threat is the frequent loss of positive pressure of the municipal supply pipeline, which risks seepage of faecally contaminated material from the local environment. Additionally, populations living in rural areas are wholly reliant on untreated water, primarily sourced from surface water such as rivers. In such rural villages, there is limited maintenance of septic tanks and other waste storage facilities, if such facilities are present at all. Thus, the combination of poor sanitation systems and continuously saturated soil creates an environment where the potential for contamination of surface and groundwater via leaking or improperly designed toilets or waste storage facilities is considerable (David Duncan and Kamal Khatri, SOPAC, personal communication).

## Recommendations for the reduction of the disease burden

In August 2012, the Fijian MOH hosted a meeting of local and international partners with the aim of developing a holistic approach to typhoid control in Fiji. Overall, it was felt that typhoid in Fiji could be addressed effectively due to the existence of a well-organised public health and healthcare system, a lack of antimicrobial resistance and controlled use of antimicrobials in the community. The current lack of *S. paratyphi* A was also seen as fortunate for controlling enteric fever as there is no vaccine for this serovar. However, the threat of antimicrobial resistance, either through importation or local evolution, led experts to suggest a sense of urgency in curbing the burden of typhoid in Fiji before the problem escalates (Parry 2004). The specific recommendations of the panel were split into two large categories: information required and actions needed.

The primary information requirements centre on collecting more data on the epidemiology of typhoid in the community (Table1). Defining potential risk factors for disease through the use of rationally designed case–control investigations was considered an important priority. Further analysis of vaccine effectiveness after the 2010 campaign was suggested in addition to the ongoing analyses (Scobie *et al*. 2014), using any available stored records, case–control investigations and through prospective evaluation of incoming typhoid patients. Exploring the viability of potential transmission pathways was also thought to be of value. Assessing growth and survival rates of *S. typhi* in the popular drink *kava* was encouraged, as even limited contamination of a *kava* bowl, combined with reasonable survival rates, could serve as a source of infection. Culturing bile aspirates from patients undergoing cholecystectomy operations to estimate the prevalence of *S. typhi* carriage in the community was also recommended (Dongol *et al*. 2012). Additionally, a serological survey of a cross-section of the population using both the Vi and Hd antigens was suggested to evaluate the rates of exposure amongst various age and ethnic groups. These measures were recommended to help inform future control policy, specifically whether a school-based vaccination strategy would be effective given the intensity of transmission and associated age distribution of culture-confirmed typhoid cases.

**Table 1:** A summary of recommendations to address information needs developed by the expert panel for the reduction and control of typhoid fever in Fiji

Information required		
Area	Recommendation	Rationale
Design and implement case–control study for risk factors	Attempt to identify important transmission routes of typhoid in Fiji and, in particular, determine likely causes for recent increase in case numbers and disparate ethnic distribution	Allow targeted interventions against salient risk factors or transmission routes
Design and implement case–control and prospective epidemiological analyses for further evaluation of 2010 vaccination campaign	Follow-up of any of the 70 000 individuals who were vaccinated in 2010; estimating the incidence in those who were not vaccinated in 2010 (<2 years of age) and compare to pre-campaign estimates to investigate evidence of herd immunity	Guide future vaccine policy: estimate effectiveness over time
Serological survey	Perform prospective or retrospective survey to quantify Vi and Hd antibody levels in various demographic subpopulations to estimate rates of exposure to *S. typhi*	Guide future vaccine policy: identify age stratified infection rates to target vaccination to appropriate age groups
Clinical audit of cases; pharmacokinetic study	Compare clinical failure or relapse rates against the weight and dosage of antimicrobial agent prescribed, and perform a pharmacokinetic study to identify an optimal dosage of ciprofloxacin in this population	Guide treatment recommendations to reduce relapse, treatment failure and gallbladder carriage in the community
Microbiological studies	Attempt to grow *S. typhi* in kava, a commonly consumed drink and hypothesised transmission route	Explore viability of this transmission route to guide prevention strategies
Gallbladder bile culture analysis	Culture bile aspirates from cholecystectomy patients to estimate the prevalence of asymptomatic chronic *S. typhi* carriage	Explore strategies for chronic carrier detection and prevention of transmission if carriage prevalence is high

Action-based recommendations (Table2) included standardising clinical case definitions for children and optimising treatment regimens for adults such that the recommended dose of ciprofloxacin is appropriate to the Fijian population. These were considered to be largely straightforward and inexpensive tasks that would substantially improve case management. Encouragement of proper hand hygiene for epidemiologically important groups such as food and drink preparers was also emphasised. Limiting the search for carriers to cholecystectomy patients and point source outbreaks was suggested to reduce the considerable workload of local microbiological laboratories that are currently required to perform stool surveys during patient contact tracing. Stool culture is an insensitive method for detecting *S. typhi* infection and carriage (Baker *et al*. 2010), and it was recommended that the practice of screening contacts as such be abandoned. Although it would be complicated by high-vaccination coverage in some areas, the panel suggested developing a serological method to screen contacts for IgG to the Vi or Hd antigen (Lanata *et al*. 1983; Chart *et al*. 2000).

**Table 2:** A summary of recommendations for action developed by the expert panel for the reduction and control of typhoid fever in Fiji

Action needed		
Area	Recommendation	Rationale
Case management and documentation	Standardise the typhoid clinical case definition for children across all hospitals, review blood culture guidelines to obtain appropriate volumes of blood from patients; ensure accurate collection of patient information such as residential address and vaccination status	Enable the ability to compare surveillance data across sites and draw reliable conclusions from epidemiological information of patients
Carrier detection	Cease the current practice of stool culture for community carrier identification and for contacts of typhoid patients; consider developing a Vi-serology based screening method for contacts	Stool culture is insensitive and eliminating it from routine practice will save resources
Environment	Work with local water and sanitation experts to optimise village water supplies and storage and treatment of household water and to upgrade toilet design to prevent environmental leakage	Faulty, poorly designed and/or lack of toilets may play an important role in maintaining typhoid transmission in Fiji
Hygiene	Develop a public information campaign to focus on handwashing for epidemiologically important groups such as food and drink preparers	Reduce the potential for point source transmission due to infected individuals and target resources efficiently
Vaccination	Consider implementation of a typhoid vaccine programme; additional analyses such as an economic evaluation and/or modelling vaccination impact should also be considered	Ensure efficient use of limited resources for vaccination by targeting appropriate subpopulations (e.g. schoolchildren, food handlers)
Long-term local capacity building	Develop a group of local individuals trained in basic epidemiology, microbiology and molecular biology	Form a hub of expertise to drive future disease control efforts
Information management	Streamline surveillance and laboratory data collection and management to ensure that data is consistent, linked with any relevant microbiology information and accessible to members of the public health community	Effectively make use of the large amount of data that is currently collected by the MOH

For a more long-term solution, it was recommended that drinking water storage and treatment at the household level, including the more reliable disinfection of urban supplies, should be optimised if the case–control investigations indicate such issues as risk factors. The panel also suggested a review of current toilet designs to reduce leakage into rain-sodden soil and to limit environmental transmission of *S. typhi*. Finally, the panel recommended developing a vaccination strategy dependent upon the serologic evaluation of exposure and further analysis of the previous vaccination campaign.

## Conclusions

Typhoid appears to be a considerable problem in Fiji, although basic epidemiological data are limited. However, the notable absence of antimicrobial resistance within the *S. typhi* population and the relative isolation of this island nation make typhoid management and control a real possibility in Fiji. The panel meeting hosted by the Fijian MOH in August 2012 discussed the current situation in the country, shared relevant experiences in typhoid control in both Fiji and other locations, consulted with local partners and developed a comprehensive set of measures for the Fijian MOH to consider implementing. A coordinated action plan, funding and support are required to sustain the current momentum in combating typhoid, particularly with the potential threat of the evolution or importation of antimicrobial-resistant strains. Finally, due to broad similarities in culture, climate and geography, a successful control programme in Fiji could potentially be used as a model for typhoid control in the Pacific region as a whole.
